# Investigating reproducibility and tracking provenance – A genomic workflow case study

**DOI:** 10.1186/s12859-017-1747-0

**Published:** 2017-07-12

**Authors:** Sehrish Kanwal, Farah Zaib Khan, Andrew Lonie, Richard O. Sinnott

**Affiliations:** 1 Department of Computing and Information Systems, The University of Melbourne, Melbourne, VIC 3010 Australia; 2 Melbourne Bioinformatics, The University of Melbourne, Melbourne, VIC 3010 Australia

**Keywords:** Reproducibility, Provenance, Workflow, Galaxy, Cpipe, Common Workflow Language (CWL)

## Abstract

**Background:**

Computational bioinformatics workflows are extensively used to analyse genomics data, with different approaches available to support implementation and execution of these workflows. *Reproducibility* is one of the core principles for any scientific workflow and remains a challenge, which is not fully addressed. This is due to incomplete understanding of reproducibility requirements and assumptions of workflow definition approaches. *Provenance* information should be tracked and used to capture all these requirements supporting reusability of existing workflows.

**Results:**

We have implemented a complex but widely deployed bioinformatics workflow using three representative approaches to workflow definition and execution. Through implementation, we identified assumptions implicit in these approaches that ultimately produce insufficient documentation of workflow requirements resulting in failed execution of the workflow. This study proposes a set of recommendations that aims to mitigate these assumptions and guides the scientific community to accomplish reproducible science, hence addressing reproducibility crisis.

**Conclusions:**

Reproducing, adapting or even repeating a bioinformatics workflow in any environment requires substantial technical knowledge of the workflow execution environment, resolving analysis assumptions and rigorous compliance with reproducibility requirements. Towards these goals, we propose conclusive recommendations that along with an explicit declaration of workflow specification would result in enhanced reproducibility of computational genomic analyses.

**Electronic supplementary material:**

The online version of this article (doi:10.1186/s12859-017-1747-0) contains supplementary material, which is available to authorized users.

## Background

**Fig. 1:**
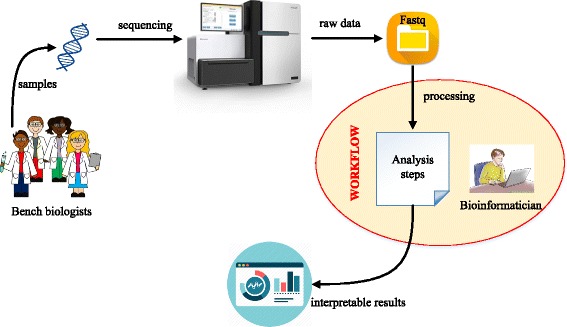
Computational bioinformatics workflows are often deployed to deal with the data processing bottleneck. A typical workflow consists of a series of linked steps that transform raw input (e.g. a fastq file produced as a result of NGS data) into meaningful or interpretable output (e.g. variant calls). Typically, these steps are performed by specific tools developed to tackle a particular functional aspect of genomic sequence analysis. Workflows can have variable number of steps depending on the type of analysis performed, hence can be simple or complex

Recent rapid evolution in the field of genomics, driven by advances in massively parallel DNA sequencing technologies, and the uptake of genomics as a mechanism for clinical genetic testing, have resulted in high expectations from clinicians and the biomedical community at large regarding the reliable, reproducible, effective and timely use of genomic data to realise the vision of personalized medicine and improved understanding of various diseases. There has been a contemporaneous recent upsurge in the number of techniques and platforms developed to support genomic data analysis [1]. Computational bioinformatics workflows are used extensively within these platforms (Fig. 1). Typically, a bioinformatics analysis of genomics data involves processing files through a series of steps and transformations, called a workflow or a pipeline. Usually, these steps are performed by deploying third party GUI or command line based software capable of implementing robust pipelines.

Significant informatics knowledge, resources, tools and expertise are required to design workflows for the analysis and interpretation of sequencing data to ultimately obtain highly specific knowledge that can be translated into clinical settings. Through efforts such as the 1000 Genomes project [1] and aligned approaches for analysis of Next Generation Sequencing (NGS) data [2], a variety of best practices for variant discovery are now available. The resulting knowledge should be unambiguous and consistent, *repeatable* (defined as a researcher redoing their own experiment/analysis in the same environment for the same result/outcome [3]), and *reproducible* (defined as an independent researcher/lab confirming or redoing that experiment/analysis, potentially in a different environment [3]) and eventually translatable into clinical (healthcare) context. Bioinformatics data analysis and variant discovery - in the clinical domain in particular - requires *provenance* [4] information to be captured for reproducibility. This provenance capture should store details of each workflow execution including the software versions, the software parameters, and the information including data produced at each workflow step [5].

The reproducibility of scientific research is becoming increasingly important for the scientific community, as validation of scientific claims is a first step for any translational effort. The standards of computational reproducibility are especially relevant in clinical settings following the establishment of Next Generation Sequencing (NGS) approaches. It has become crucial to optimize the NGS data processing and analysis to keep at pace with the exponentially increasing genomics data production. The ability to determine DNA sequences has outrun the ability to store, transmit and interpret this data. Hence, the major bottleneck to support the complex experiments involving NGS data is data processing instead of data generation. Computational bioinformatics workflows consisting of various community generated tools [6] and libraries [7, 8] are often deployed to deal with the data processing bottleneck.

Despite the large number of published literature on the use and importance of -omics data, only a few have been actually translated into clinical settings [9]. The committee on the review of -omics-based tests for predicting patient outcomes in clinical trials [10] attributed two primary causes; inadequate design of the preclinical studies and weak bioinformatics rigour, for this limited translation. The scientific community has paid special attention with respect to benchmarking -omics analysis to establish transparency and reproducibility of bioinformatics studies [11]. Nekrutenko and Taylor [12] discussed important issues of accessibility, interpretation and reproducibility for analysis of NGS data. Only ten out of 299 articles that cited the 1000 Genomes project as their experimental approach used the recommended tools and only four studies used the full workflow. Out of 50 randomly selected papers that cited BWA [13] for alignment step, only seven studies provided complete information about parameter setting and version of the tool. The unavailability of primary data from two cancer studies [14] was a barrier to achieve biological reproducibility of claimed results.

Ioannidis et al. [15] attributed unavailability of data, software and annotation details as reasons for non-reproducibility of microarray gene expression studies. Hothorn et al. [16] found that only 11% of the articles conducting simulation experiments provided access to both data and code. The authors reviewing 100 Bioinformatics journal papers [17] claimed that along with the textual descriptions, availability of valid data and code for analysis is crucial for reproducibility of results. Moreover, the majority of papers that explained the software environment, failed to mention version details, which made it difficult to reproduce these studies.

To facilitate genomic data analysis, various Workflow Management Systems (WMS) are specifically designed and available to meet the challenges associated with such data [18]. Typically WMS are designed to support the automation of data-driven repetitive tasks in addition to capturing complex analysis processes associated with data processing steps. Having sufficient provenance information plays a major role in understanding data processing steps incorporated in a workflow and ensures the consistency of the results with the known (current) best practice [19]. Ludäscher et al. [20] reviewed common requirements of any scientific workflow, most of which (such as data provenance, reliability and fault-tolerance, smart reruns and smart semantic links) are directly linked to provenance capture. In addition to *workflow evolution* [21], *prospective* (defined as the specification of the workflow used in an analysis) as well as *retrospective* (defined as the run time environment of an execution of the workflow in an analysis) provenance [22] was identified as an essential requirement for every computational process in a workflow to achieve reproducibility of a published analysis and ultimately accountability in case of inconsistent results. Several provenance models have been proposed and implemented to support retrospective and prospective provenance [23–25] but these are seldom used by WMS used in genomic studies. Despite high expectations, various existing WMS [26–30] do not truly preserve all necessary provenance information to support reproducibility - particularly to the standards that might be expected for clinical genomics.

The inability to reproduce and use exactly the same procedures/workflows means that considerable effort and time is required on reproducing results produced by others [12, 16, 17, 31]. At present the consolidation of expertise and best practice workflows that support reproducibility are not mature. Most of the time, this is due to the lack of understanding of reproducibility requirements and incomplete provenance capture that can make it difficult for other researchers to reuse existing work. The sustainability of clinical genomics research requires that reproducibility of results goes hand-in-hand with data production. We, as the scientific community, need to address this gap by proposing and implementing practices that can ensure reproducibility, confirmation and ultimately extension of existing work.

Towards these objectives, this work contributes to the classification of the available approaches to workflow definition. Further we identified assumptions implicit in the investigated representative workflow definition approaches. In a previous study [19] that investigated challenges of large scale biomedical workflows, we had proposed, for reproducibility of science, one of the most important steps is to record summary of assumptions followed in the workflow. To address the aforementioned assumptions, we propose a generalised set of recommendations to researchers, which can be used to mitigate the challenges associated with incomplete documentation of an analysis, hence supporting reproducibility.

We have implemented a complex yet widely used exemplar variant calling workflow [32] using three approaches to workflow definition (detailed in section Approaches to workflow definition and implementation) to identify *assumptions* implicit in these approaches. The intricate underlying details associated with workflow implementation, considered needless to be stated, lead to various factors often hidden from the user. In this study, we refer to such factors as assumptions and investigate workflow definition approaches to highlight these assumptions that lead to limited or no understanding of reproducibility requirements due to lack of documentation and comprehensive provenance trace. Our study proposes a generalised set of recommendations for bioinformatics researchers to minimise such assumptions hence support reproducibility and the validity of genomic workflow studies.

## Methods

We have implemented an end-to-end complex variant calling workflow based on the Genome Analysis Tool Kit (GATK) [32] recommended best practices, using three different exemplars to workflow definition approaches: Galaxy [27], Cpipe [33] and CWL [34]. The GATK best practice variant discovery workflow was selected because it provides clear, community advocated step-by-step recommendations for executing variant discovery analysis with high throughput sequencing data on human germline samples. The next section will broadly discuss the classified approaches typically followed for workflow design and implementation and justify our choices for the systems used in this case study.

### Approaches to workflow definition and implementation

In this section, we classify approaches to workflow definition and implementation into three broad categories. Specifically, these categories have been devised on the basis of the current most common practices in the computational genomic analysis such as the pre-built pipelines driven by individual laboratories or groups; pre-configured graphical interface based workbenches and standardized workflow description implementations. This categorisation provides the basis for the selection of exemplar workflow systems investigated in this study.

#### Bioinformatics specific pre-built pipelines

Several automated bioinformatics-specific pipelines such as Cpipe [33], bcbio-nextgen [35] and others [36, 37] have been developed using command line tools to support genomic data analysis. These pipelines are driven and supported by individual laboratories, which have developed customized pipelines for processing data. This approach has resulted in considerable variability in the methods used for data interpretation and processing. The advantages of these pipelines include editing pipelines on remote servers without requiring access to GUI so that they are easily administered through source code management tools [38]. However, the command line based pipeline frameworks such as bpipe [39], Snakemake [38] and Ruffus [40] used to develop these systems are not flexible enough to support integration of new user-defined steps and analysis tools. Working with such systems requires expertise with command-line programming and broad computational knowledge as these systems extensively use individual scripts to tie together different components of the pipelines. These scripts control variables, dependencies and conditional logic for the efficient processing of the data and hence are often difficult to be reproduced. These systems assume the provision of the same physical or virtualized infrastructure used to run the initial analysis, including scripts, test data, tools, reference data and databases. The implementation overheads of such pipelines include configuration and installation of software packages, parameter setting alteration, debugging and input/output interfacing. In summary, considerable effort and excessive amount of time is required to create, understand and reproduce a ready-to-use pipeline.

#### Graphical User Interface (GUI) based integrative workbenches

To tackle some of the challenges of pipelines created using command-line interface based pipeline frameworks, workbenches [20, 27, 30, 41–43] have been developed to allow easy and customised workflow definitions using a GUI. Few of the referenced workbenches assist researchers to specify the goals, requirements and constraints for workflows using semantic reasoning, hence automating and validating complex data processing tasks [44]. Semantic workflow management systems support setting up an analysis by providing parameter preferences, alternate software tools and relevant datasets built upon the analytic constraints articulated by the user resulting in access to domain specific expertise for workflow design and configuration [45]. The semantic descriptions expect complex validation rules for input and output data objects, hence haven’t been widely adopted because of the complications involved in modelling systems, the rapid evolution of semantic web services and the majority of existing approaches adopting a non-semantic approach [46]. GUI based workbenches are typically expected to be highly featured and pre-configured with the modular tools to offer interactive design to a wide range of audience with varying degree of expertise. These often include reference datasets and configuration settings to aid users in designing automated and robust pipelines that provide managed access to a library of systems with abstraction of the interaction layer and equipped with a workflow layer that captures tool versions and parameter information. The GUI workbenches can be easily used with already existing tools but adding a new tool (plug in) or executable wrapper requires an in-depth familiarity with acceptable input file types, parameter settings, exception handling and resource management.

However, these systems do not require any local installations for analysis tools and customisation of analysis environment; hence have lower infrastructure maintenance costs. On the other hand, the availability of external services and customised tool repositories poses a risk to reproducibility as it will be impossible to reproduce a workflow created using a service which has been changed or is no longer available. Similarly workflows implemented on one system may not be reproducible when imported into another system due to incompatibility between locally customised environments.

#### Standardized approach to workflow definition

The heterogeneity in the field of in silico genomic analysis has motivated researchers to work towards standardized workflow description languages such as Common Workflow Language (CWL) and Workflow Definition Language (WDL) [47]. A variety of software platforms, such as individual workstation to high performance computing platforms (cloud, grid or cluster), can be deployed to implement these systems. Such systems provide a formal specification covering all aspects of a workflow implementation including tool versions, input data, customizable parameter settings and the workflow runtime environment that is completely independent of the underlying compute environment. Such approaches provide software specifications that help researchers define and implement portable, easy to use and reproducible workflows. These specifications aim to describe a data and execution model allowing users to have full control for creating and running the workflow by explicitly declaring the relevant environment, resources and other customizable settings in the specification.

### Case study

To comprehensively understand and identify assumptions that are implicit in the approaches detailed in section Approaches to workflow definition and implementation, we consider the impact of *reproducibility* requirements on real-world genomic systems. To this end, we have implemented an end-to-end complex variant calling workflow based on the GATK recommended best practices, using three exemplar workflow definition approaches: Galaxy, Cpipe and CWL as major representatives of the existing workflow systems used to analyse genomics data. Galaxy, an example graphical user interface based integrative framework, is an open source, web-based platform for accessible, reproducible and transparent genomics research. It supports degrees of workflow provenance with focus on assisting the capture of computational methods that are used. Cpipe an exemplar of bioinformatics specific prebuilt pipelines, adopted by Melbourne Genomics Health Alliance, uses a programmatic approach which effectively includes everything necessary for reproducing a given genomic analysis, provided the same physical or virtualized infrastructure used to run the initial analysis, including scripts, test data, tools, reference data and databases. CWL, an exemplar of declarative approach to workflow definition, enables full control to users for creating and running the workflow using a specification which is a standard descriptor for relevant environment, command line tools and other customizable settings; hence making very few internal assumptions about the requirements of the workflow. We have restricted our case study to have one representative system from each category defined in section Approaches to workflow definition and implementation.

We used chromosome 21 data for this study. It was extracted from The Genome in a Bottle dataset NA12878 which is widely used as test data because of the pre-existing and extensive analysis done on this sample and the agreed variant call truth set [48] that can be used for comparative evaluation. Other files required for the GATK workflow execution include human reference genome (hg19.fasta) and the known variant (vcf) reference files (available at https://github.com/skanwal/GATK-CaseStudy), which were obtained from the resource bundle provided by the Broad Institute.^1^

#### Workflow enactment using the selected systems

This section details the enactment process of the GATK variant calling workflow using three exemplar workflow definition approaches. We elaborate the assumptions implicit in each approach while dealing with various workflow features.

##### Cpipe

Cpipe belongs to the category of bioinformatics specific prebuilt pipeline. It was deployed on the National eResearch Collaboration Tools and Resources (NeCTAR) research cloud.^2^ The instructions on the official Cpipe GitHub page [49] were followed to setup the pipeline.

- The instance launched for executing cpipe had 16cores and 64GB RAM. The automated mechanism to document and convey compute requirement for a specific customized analysis is not defined. Rather the prebuilt pipelines presume availability of sufficient compute power to deal with data intensive steps such as sequence alignment.
- To cater for the storage requirement of the pipeline, 1000GB volume was mounted to the cloud instance. Similar to compute requirement, there is no automated mechanism for explicitly recording storage requirement. As the genomic sequence analysis involves dealing with huge input and intermediate datasets (including whole genome reference data), the prebuilt pipelines assume availability of sufficient capacity to deal with data storage requirements.
- The installation script provided with cpipe compiled tools such as BWA and downloaded databases such as Variant Effect Predictor (VEP) and human reference sequence files. The prebuilt pipelines connect to online resources to download and compile tools and reference datasets used in the analysis. FTP clients and SSH transfer tools are used for moving datasets over distributed resources. The availability of high performance networking infrastructure is assumed to move bulk data using wide area network (WAN).
- The base software dependencies for underlying programming frameworks such as Java and Python were required to execute tools in cpipe. The prebuilt pipelines assume that users are responsible to solve base software dependencies for the pipeline; otherwise the pipeline would fail to execute.
- Cpipe requires downloading and pre-processing the reference data set to generate secondary files since the indexing step is not explicitly defined as part of the pipeline but included in a separate script. The pre-built pipelines expect users to perform pre-processing steps and hence assume availability of input data files to be made available before execution of the pipeline.
- Cpipe uses a copyrighted tool, ANNOVAR, for annotating variant calls. The prebuilt pipelines deploying copyrighted or proprietary tools, instead of open source software, assume users to ensure availability of all such licensed resources.
- Cpipe requires a specific directory structure in order to execute the analysis on any sample. As the prebuilt pipelines are customized to support explicit analysis requirements, these assume availability of a specific analysis environment with a set directory structure, having tools and datasets appropriately located to support seamless execution of the pipeline. Files and tools are expected to be placed according to particular file system hierarchy since paths are hard coded in the scripts.

##### Galaxy

Galaxy was selected from the category of GUI based Integrative workbenches to implement GATK variant calling workflow (Fig. 1-Additional file 1).^3^ The Genome Virtual Laboratory (GVL) [50] was used for launching a pre-configured Galaxy instance on an OpenStack-based cloud environment.

**Fig. 2:**
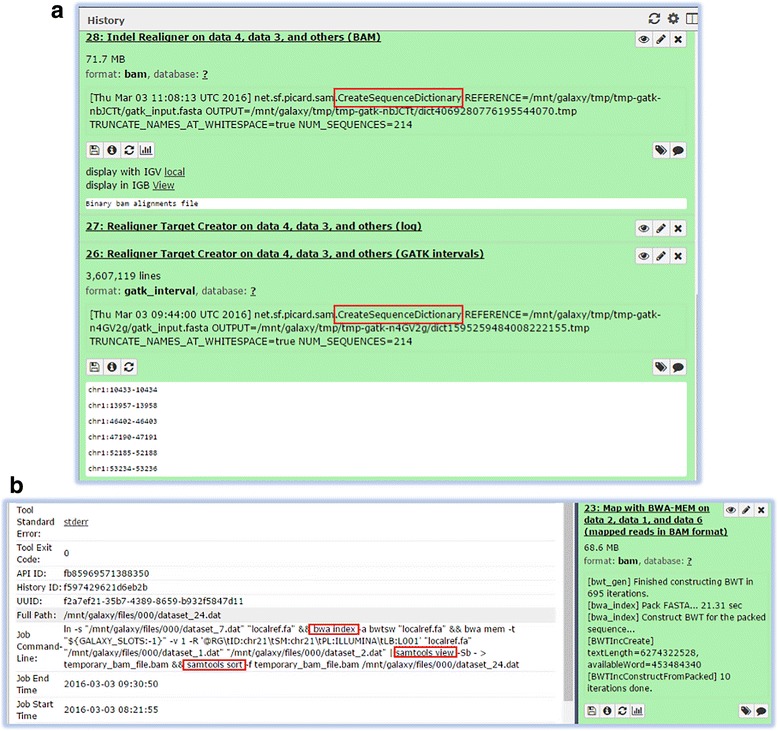
Screenshots of the Galaxy interface showing (**a**) A temporary sequence dictionary file creation using CreateSequenceDictionary as part of RealignTargetCreator and IndelRealigner step and (**b**) “Map with BWA-MEM” step combining indexing reference data, SAM to BAM conversion and sorting of the resultant aligned (BAM) file

- Specifically, a GVL 8-core cloud instance with 32GB RAM was launched to provision a fully configured Galaxy for the analysis of NA12878. Similar to prebuilt pipelines, GUI based workbenches also assume the availability of sufficient compute power to process data, hence are user dependent for the provision of these resources. Galaxy workbench lacks a prior check to ensure availability of sufficient compute resource.
- A 1000GB volume was mounted to the GVL instance launched. GUI based workbenches require the user to provide sufficient storage capacity to deal with the data storage, hence the workflows built using these workbenches have little or no explicit declaration of such requirements.
- Chromosome 21 fastq files, known variant vcf databases and hg19 reference sequence fasta file (provided in the supplementary material) were uploaded to Galaxy. Galaxy uses inbuilt reference files if not provided by users but other databases are expected to be provided by users. Even if a complete workflow built on such systems is published, not only is the provision of input data the user’s responsibility but these systems also assume the availability of supporting data (such as reference sequence and variant databases) to generate results.
- During implementation, it was observed that Galaxy automatically performs certain steps without explicitly declaring them such as indexing the provided reference genome, creating index files for the BAM output file (using Picard [51] mark duplicates), generating a temporary reference sequence dictionary as part of the local realignment steps and creating a fasta index file for GATK tools (Fig. 2). GUI based workbenches simplify the interface and facilitate user by hiding the underlying details from the user. This results in an inability to replicate or reproduce the same workflow due to incomplete or implicit documentation.
- In Galaxy, reference sequence indexing, SAM to BAM conversion and sorting the resulting BAM file is embedded in the alignment step and does not appear in the final workflow diagram. The visual dataflow diagram produced by such systems is assumed to be a complete picture of the processes carried out during a workflow execution. The absence of an entire step of pre-processing, processing or post processing data from the workflow details especially from visual representation leads to incomplete workflow knowledge when attempted to be reproduced.
- The Galaxy toolshed is populated with tools configured using XML specifications requiring technical and extensive programming expertise to write XML configuration files for the tool versions that are not available in the toolshed. A Galaxy workflow requires availability of uniform toolsheds across Galaxy instances, therefore a workflow created using particular tool versions on one instance will fail to execute on instances with a toolshed supporting different tool versions. This renders it inflexible, static and a challenge to reproducibility. The workflow developers assume uniformity of tool repositories across different instances of a workbench. Hence, workflows created using GUI based workbenches are tied to specific versions of tools used to declare the workflows and the absence of these specific tool versions will result in failed execution of workflow.

##### CWL

CWL aims for a standardized approach to workflow definition. It was cloned and installed following the instructions from the GitHub repository [52].

- A reference implementation of CWL designed specifically for Python 2.7 was cloned and installed following the directions from the GitHub repository manual.^4^ The availability of the specific underlying language and its particular version for reference implementation (Python in this case) is assumed for successful installation and functioning of the reference implementation.
- Working with CWL was challenging as compared to Cpipe and Galaxy because it is an ongoing, constantly developing community effort and tool wrappers for most of the required tools for this study were not available. Implementing the GATK workflow in CWL required the knowledge of Yet Another Markup Language (YAML) and JavaScript Object Notation (JSON) for development of a number of CWL definition files including YAML tool wrappers, JSON job files containing the input parameters and YAML test files for conformance tests (Fig. 2-Additional file 1).^5^ It is assumed that any user wanting to utilise these definition files along with the workflow definition should have basic understanding of YAML and JSON. In addition, if a newer version or different tool is required for any step, the user is expected to develop the definition files for which in depth knowledge of underlying languages is required. Therefore, the standardized approaches on the one hand provide users with the freedom to declare every aspect of the workflow but on the other hand assume the implicit knowledge of underlying languages leading to steep learning curves for naïve users.
- The workflow implementation used tools such as BWA, GATK and Picard Toolkit which were provided through container-based Docker^6^ images including all required software packages. This step required installation of Docker which again was assumed to be available on the system executing the workflow. Although CWL encourages use of Docker, it also facilitates the local installation of required tools which should not be preferred as it will lead to localised solutions that fail to execute elsewhere. In both cases, certain assumptions were made regarding availability of the underlying tool and their link with the tool definition. Hence, the standardized approaches despite making efforts to explicitly declare every step of the workflow assume the underlying software availability for enactment of a workflow which is not always the case.
- As genomic workflows usually involve working with large datasets, the availability of compute and storage resources is assumed to be managed by users to successfully enact workflows.

## Results and discussion

The expectation for science to be reproducible is considered fundamental but often not tested. Every new discovery in science is built on already known knowledge, that is, published literature acts as a building block for new findings or discoveries. Using this published literature as a base, the next level of understanding is developed and hence the cycle continues. Therefore, if we cannot reproduce already existing knowledge from the literature, we are wasting a lot of effort, resources and time in doing potentially wrong science [53] resulting in “reproducibility crisis” [54]. If a researcher claims a novel finding, someone else, interested in the study, should be able to reproduce it. Reports are accumulating that most of the scientific claims are not reproducible, hence questioning the reliability of science and rendering literature questionable [55, 56]. The true reproducibility of experiments in different systems has not been investigated rigorously in systematic fashion. For computational work like the one described in this paper, reproducibility not only requires an in depth understanding of science but also data, methods, tools and computational infrastructure, making it a non-trivial task. The challenges imposed by large-scale genomics data demand complex computational workflow environments. A key challenge is how can we improve reproducibility of experiments involving complex software environments and large datasets. Although this question is pertinent to scientific community as a whole [57], here we have focused on genomic workflows.

Reproducibility of an experiment often requires replication of the precise software environment including the operating system, the base software dependencies and configuration settings under which the original analysis was conducted. In addition, detailed provenance information of required software versions and parameter settings used for the workflow aids in the reusability of any workflow. Provenance tracking and reproducibility go hand in hand as provenance traces contribute to make any research process auditable and results verifiable [58]. The variant calling workflows (as our case study) result in genetic variation data that serves to enhance understanding of diseases when translated into a clinical setting resulting in improved healthcare. Keeping in view the critical application of the data generated, it is safe to state that entire process leading to such biological comprehensions must be documented systematically to guarantee reproducibility of the research. However a generalised set of rules and recommendations to achieve this is still a challenge to be met as workflow implementation, storage, sharing and reuse significantly varies depending on the choice of approach and platform used by the researcher. A common phenomenon to every approach however is ‘workflow decay’ [59] caused by the factors such as the evolution of technical environment used to implement a workflow, updates in the state of external factors such as databases and unavailability of third party web resources. Our study contributes to understanding the requirements of reproducibility of genomic workflows by investigating a set of assumptions evident from practical implementation of the case study and providing standardised recommendations for computational genomic workflow studies.

Owing to the production of exceptional amounts of genomics data, a typical human exome sequence analysis (for example the current case study) would require a terabyte of storage and up to 64GB RAM of compute power. As the computational dependencies of workflows have grown complex from simple batch execution to distributed and parallel processing, researchers should document and provide the amount of storage and compute power required by a workflow to run successfully. Long term reproducibility of scientific results can be hard to achieve if the appropriate resources required to reproduce the workflow are not fully declared. Apart from declaration of compute and storage resources required to successfully execute a workflow, comprehensive efforts by workflow developers could result in better management of dependencies. A tool or a workflow built on a specific computing platform requires the details of the exact version of the underlying base software to execute successfully. One example is a requirement of a particular version of Java (1.8) to execute tools from GATK or Picard toolkit used in a workflow. The absence of such information about the base software requirements such as Java or Python would result in at least one unsuccessful execution of the workflow. We recommend workflow developers devise a mechanism (e.g. provide a script) that should implement checkpoints to analyse the suitability of computing platform before the execution attempt. This will ideally guide the researchers trying to reproduce a workflow who otherwise would waste considerable time tackling the *‘dependency hell’*. The burden obviously shifts to the workflow developers but in the longer run, it would be helpful to declare and document the very basic information, which is considered too obvious to state.

Genomic data analysis has grown complex with the increased involvement of customized scripts and online resources needed to carry out difficult tasks, increasing both the technical knowledge required and the chance that something will break. One of the major reasons for non-reproducibility of workflows is use of volatile third party resources such as databases, tools or websites [59]. Many workflows cannot be run because the third party resources they rely on are no longer available and the results could only be reproduced using the specific version of the software, hence rendering workflows unusable. These factors can be considered out of control of the researchers as every time an analysis is repeated, it may assume that the system it is being reproduced on comes preconfigured with all the workflow dependencies. Also, the download of large genomic datasets from the third party online resources demands users ensure availability of high performance networking infrastructure on their part. Volatile third party resources is an open end problem to which several solutions have been proposed, such as alternative resources or local copy of the resource, to mitigate the consequences [60]. However, we believe that alternative resources might not result in the same output, hence a barrier to reproducibility of results [19]. The services hosting third party resources are generally in no agreement to continuously supply these resources. Even the most sophisticated and widely used technologies such as container based approaches require connection to the network and online resources at least once for building of the required software components.

Third party services such as copyrighted or proprietary resources should be avoided in research involving use of genomic datasets as they can result in an inability to access original resources or tools, overshadow the ramifications of the research and halt reproducibility. The possible solutions to reproduce the research involving such tools can be through buying or re-implementing these software, which is often not a realistic expectation. Instead the community should push forward to work towards open source software and collaborative science [61], which makes it easier to communicate and access scientific knowledge. The efforts such as Centre for Open Science^7^ are working towards encouraging openness and reproducibility of scholarly research, hence accelerating scientific progress.

Additionally, explicit requirements for specific analysis environment, e.g. hard coded paths and names embedded in source code, should be avoided in the pipeline definition. In our case study, creation of an analysis environment with a particular directory and file naming convention was required by Cpipe to execute the workflow successfully [33]. From our experience, we recommend that this should not be a rule as it adversely affects the portability of the workflow. An extra responsibility on a researcher reproducing someone else’s workflow is to define the analysis environment and related parameters. We recommend avoiding the hard coding file names, absolute file paths, host names, user names and IP addresses. Workflow developers should ensure their workflows are independent of a specific analysis environment to allow their workflows to be more readily executable.

In principle, workflow management systems such as Galaxy use a publicly shared repository for published tools and workflows. In practise this is a challenge, as there are many ways to set up the analysis to begin with. Galaxy allows the users to choose the computing platform such as centralised public galaxy, galaxy on cloud or as a localised instance. There are more than 80 publically shared galaxy servers^8^ each containing different toolsets. Workflow developers can create a workflow using their localised instances and later publish these workflows assuming uniformity of tool repositories across different platforms. This can result in static and inflexible solutions, hence challenging to be reproduced as it assumes uniformity of repositories across different platform instances. The workflow developers are recommended to ensure the availability of the tools used in the workflow implemented on local instances of any workflow management system. These tools should either be shared via repositories associated with a certain workflow system or using open source code sharing solutions e.g. through a git repository. The repository maintainers should make the process of adding tools to centralized repositories straightforward and easy to implement. This would result in cost effective analysis encouraging researchers to reuse the resources provided instead of reinventing the wheel.

Input such as sequencing reads in FASTA files and reference datasets play a major role to enable reproducibility of genomic workflows and ultimately achieve repeatable results. Even in the case where the user has comprehensive understanding of the workflow analysis, absence of input data annotations hinders the successful execution of the workflow. Analysis tools usually require strict adherence to file formats (e.g. reference sequence should be a single reference sequence in FASTA format or the names and order of the contigs in the reference used must exactly match that of one of the official reference canonical orderings). This demands providing access to primary data used in the analysis. However, a major implication of this idea lies in the security and ethical consideration of genomics data. The community needs to address this issue by providing secure controlled access to sensitive genomic data. Also, the size of the genomic datasets can be a problem in sharing the datasets and providing them to workflow specifications. In such cases, where it is not possible to package or share datasets with the workflow, comprehensive annotations will assist researchers to decide on the appropriate datasets for the workflow. Public repositories^9^^,^ 10 and resources can also be used to archive, preserve and share genomic datasets.

With ever evolving repositories, services, tools and data, workflow specification alone is rarely sufficient to ensure reproducibility and reusability of scientific experiments, resulting in workflow decay. One way to avoid the workflow decay is to provide complete provenance capture including annotations for every process during workflow execution, the parameters and links to third party resources including data and external software services. This information should be available with the published workflow. The relevant parameter setting for each tool used in an analysis is also essential to ensure reproducibility of results hence should be provided with the workflow. Alternatively workflow developers should package all associated tools when the workflow is published. Workflows should be treated as first class data objects [62] and container technologies such as Docker, OpenVZ^11^ or LXC^12^containers should be used to package the environment and configuration together.

Approaches such as CWL utilise Docker containers, work on the principles of comprehensive declaration and make minimal internal assumptions about the precise software environment, base software dependencies, configuration settings, alteration of parameters and software versions. Such approaches aim to build flexible and customized workflows including intricate details of every process in a workflow such as requirement declarations for the runtime environment, data and metadata, input and output parameters and command-line executable. This results in archiving of the entire framework of the software environment that can be re-established to support reproducibility. However, working with this kind of approach is not an easy task and requires lots of time, efforts and substantial technical support (in our case study this was provided by the CWL team) to first learn the principles of the language and then coding to implement system configuration of a complex genome analysis workflow.

**Fig. 3:**
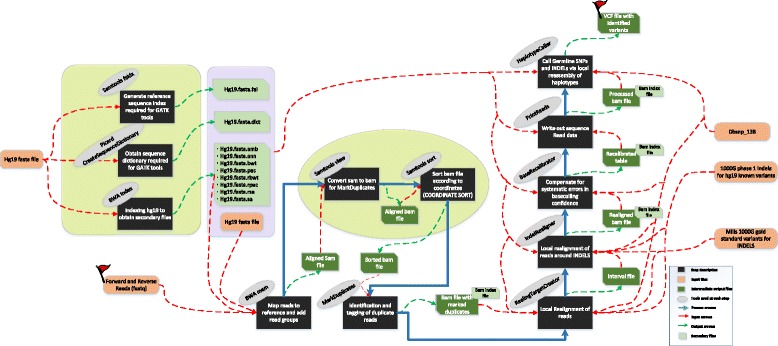
Graphical representation of the GATK workflow representing artefacts and information necessary to be captured as part of workflow execution. The description of main steps is depicted in the *black rectangles* whereas the tools responsible to carry out the steps are shown in *grey ellipses*. Input and reference files (*brown rounded rectangles*) are shown separately and labelled by the dataset name. The primary and secondary output files (if any) are shown in *dark* and *light green snip diagonal corner rectangles* respectively. The input and output data flow for each workflow step is demonstrated through *red* and *green dotted arrows* respectively. The connection between processes in a workflow is represented by *blue solid arrow*. The *yellow highlighted parts* of the workflow are the pivotal processes not explicitly declared in Galaxy and Cpipe. The *red flag* highlights the main input and final output for the workflow

**Fig. 4:**
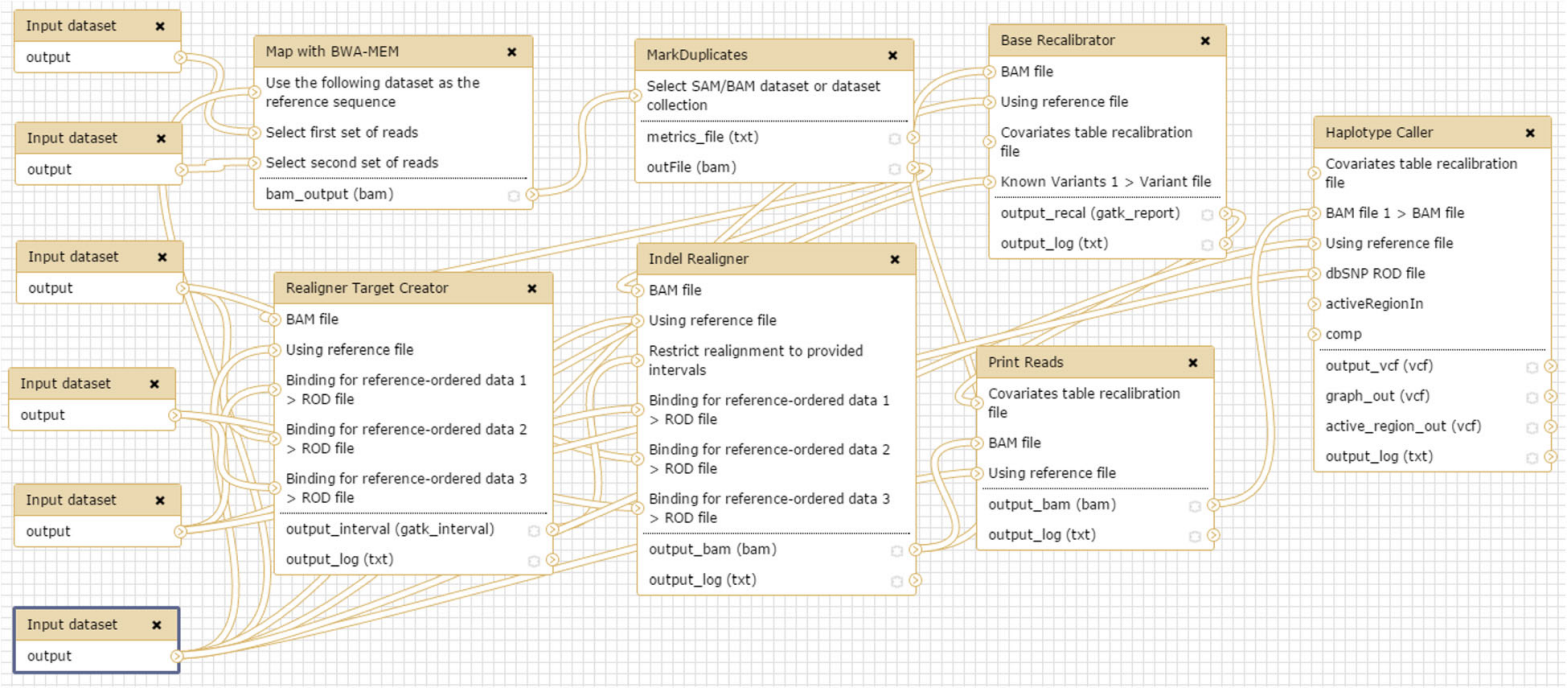
The variant calling workflow representation in Galaxy

Hence, the details vital to reproducibility of any computational genomic analysis should be completely documented to ensure capture of critical provenance information. From our experience gained from this study we posit that the workflow developers along with other mechanisms should collectively document the important pieces of information through graphical representation of the workflow as indicated in Fig. 3. The flowchart in the figure can be used as a model to record a high level representation of the underlying complex workflow. It is a blueprint containing all the artefacts including tools, input data, and intermediate data products, supporting resources, processes and connections between these artefacts. To re-enact any workflow the users should be directed to explicitly understand and declare all the requirements mentioned in such workflow representation. The proposed representation of the variant calling workflow shown (Fig. 3) contains all the necessary artefacts needed to support reproducibility requirements and provenance tracking across the platforms. The concept of visual representation of the workflow is implemented in only a few GUI based workbenches [27, 30, 63] but such high level representation depicts an inadequate illustration of the analysis as evident from Fig. 4.

During this study we observed that the ultimate Galaxy workflow diagram does not state the utilization of some tools such as BWA Index, SAMtools View, SAMtools Sort, SAMtools Faidx and Picard CreateSequenceDictionary. Therefore the incomplete Galaxy workflow diagram (Fig. 4) is challenging to be reproduced on other platforms, as necessary information about each step is not recorded. Hence, platforms making assumptions about some aspects of a workflow without documenting them as part of final workflow diagram result in incomplete understanding of the reproducibility requirements.

The workflows used to implement biomedical data analyses have grown complex [64] making it difficult to understand and reproduce such experiments. A graphical representation (Fig. 3) allows visualization of multiple aspects of workflow definition and implementation including data manipulation and interpretation. Enabling simplicity by representing complex workflows in human readable formats can significantly reduce the complexity of such analyses through improved understanding. As the studies involving complex analysis tasks encompass human judgments, it is important that the research community works in this direction to help researchers transfer their knowledge and expertise using proposed rich and easy to create representations. Further, the proposed human readable description (along with the machine readable ones) can help identify bottlenecks in the analysis and ultimately accelerate reproducibility of data driven sciences.

## Conclusion

Reproducibility of computational genomic studies has been considered as a major issue in recent times. In this context, we have characterised workflows on the basis of approach used for their definition and implementation. To evaluate reproducibility and provenance requirements, we implemented a complex variant discovery workflow using three exemplar workflow definition approaches. We identified numerous implicit assumptions interpreted through the practical execution of the workflow, leading to recommendations for reproducibility and provenance, as shown in Table 1.

**Table 1:** Summary of assumptions (detailed in section Workflow enactment using the selected systems) and corresponding recommendation for reproducibility

Assumptions	Recommendations
Availability of sufficient storage and compute resources to deal with processing of big genomics data	Workflow developers should provide complete documentation of compute and storage requirements along with the workflow to achieve long-term reproducibility of scientific results.
Availability of high performance networking infrastructure to move bulk genomics data	Considering the size and volume of genomic data, researchers reproducing any analysis should ensure that an appropriate networking structure for data transfer is on hand
The computing platform is preconfigured with the base software required by the workflow specification	Workflow developers should provide a mechanism with check points to ensure compatibility of the computing platform deployed by a researcher to reproduce the original analysis
Users are responsible to ensure access to copyrighted or proprietary tools	Community should encourage work leveraging open source software and collaborative approaches thereby avoiding use of copyrighted or proprietary tools
Analysis environment with a particular directory structure and file naming conventions is setup before executing the workflow	Workflow developers should avoid hardcoding environmental parameters such as file names, absolute file paths and directory names that would otherwise render their workflow dependent on a specific environment setup and configuration
Appropriate datasets are used as input to the tools incorporated in the workflow	As bioinformatics analysis tools require strict adherence to input or reference file formats, data annotations and controlled access to primary data can ultimately help reproduce the workflow precisely
Users will have a comprehensive understanding of the analysis and the provided information (in the form of incomplete workflow diagram) is sufficient to convey high level understanding of the workflow	Workflow developers should provide a complete data flow diagram serving as a blue print containing all the artefacts including tools, input data, intermediate data products, supporting resources, processes and the connection between these artefacts
Availability of specific tool versions and setting relevant parameter space	Tools should either be packaged along with the workflow or made available via public repositories to ensure accessibility to the exact same versions and parameter settings as used in the analysis being reproduced, hence supporting flexible and customizable workflows.
Users to have proficient knowledge of the specific reference implementation	This factor might be considered out of control of the workflow developers but detailed documentation of the underlying framework used and community support can help in overcoming the associated learning curve

Workflows are often (typically!) dependent on the replication of complex software environments necessitating substantial technical support to reproduce the configuration settings required for the analysis. This varies depending on the different approaches taken to workflow design and execution. The assumptions followed in each approach are one of the reasons for this heterogeneity that subsequently results in incomplete documentation of workflow requirements. Our case study illustrates the variability in workflow implementations based on the platform selected that can impact on crucial requirements for reproducibility and provenance that is currently missing from workflows. Ensuring reproducibility is highly dependent on the efforts of researchers to convey their analysis in a way that is comprehensive and understandable. We posit that adhering to proposed recommendations along with an explicit declaration of workflow specification would result in enhanced reproducibility of computational genomic analyses. The graphical representation proposed in this study can potentially be translated using the available community accepted standards for provenance [65] and tested across different platforms to generalise it for further extension to other workflows. In future, it would be interesting to extend this case study with other workflow systems such as Wings, Kepler, WDL, VisTrails and Taverna to analyse the reproducibility and provenance requirements, hence potentially updating the recommendations if any assumption specific to these systems is identified.

## Additional file

Additional file 1: This file contains details about enactment of GATK variant calling workflow using Galaxy, Cpipe and CWL﻿. (PDF 585 kb)

## Abbreviations

CWL: Common Workflow Language
GATK: Genome Analysis Toolkit
GUI: Graphical User Interface
WMS: Workflow Management System

## Footnotes

1
www.broadinstitute.org/gatk/guide/article.php?id=1213

2
https://nectar.org.au/research-cloud/

3
https://github.com/skanwal/GATK-CaseStudy/blob/master/SupplementaryMaterial/AdditionalFile1.pdf

4
https://github.com/common-workflow-language/cwltool

5
https://github.com/skanwal/GATK-CaseStudy/blob/master/SupplementaryMaterial/AdditionalFile1.pdf

6
https://www.docker.com/

7
https://cos.io/

8
wiki.galaxyproject.org/PublicGalaxyServers

9
http://www.data.cam.ac.uk/funders

10
http://www.nature.com/sdata/policies/repositories

11
https://openvz.org/Main_Page

12
https://linuxcontainers.org/
