# Supplementary material for: Investigating reproducibility and tracking provenance – A genomic workflow case study
Source: BMC Bioinformatics. 2017 Jul 12;18:337. doi: 10.1186/s12859-017-1747-0 (PMC5508699; doi:10.1186/s12859-017-1747-0)
Supplement: Additional file 1: — This file contains details about enactment of GATK variant calling workflow using Galaxy, Cpipe and CWL﻿. (PDF 585 kb) [file 12859_2017_1747_MOESM1_ESM.pdf]

This document contains details about execution of the GATK variant calling workflow and depicts the difference between the visual appearances of the three approaches adopted for the case study under study.

Galaxy:

Figure 1 is the snapshot of the Galaxy environment displaying toolshed populated with preconfigured tools necessary for our case study workflow implementation. These pre-existing tools were connected on the workflow canvas, which allows users to drag and drop the required tools to construct a workflow. The input files and parameters of the tools (incorporated into the GATK workflow) were customized using the workflow design canvas. All the tools were executed using default settings and no manual alteration of parameter settings was made except for providing read group header to BWA mem and known variant reference files to the tools. The workflow was saved in the form of a Galaxy 'history' after successful execution.

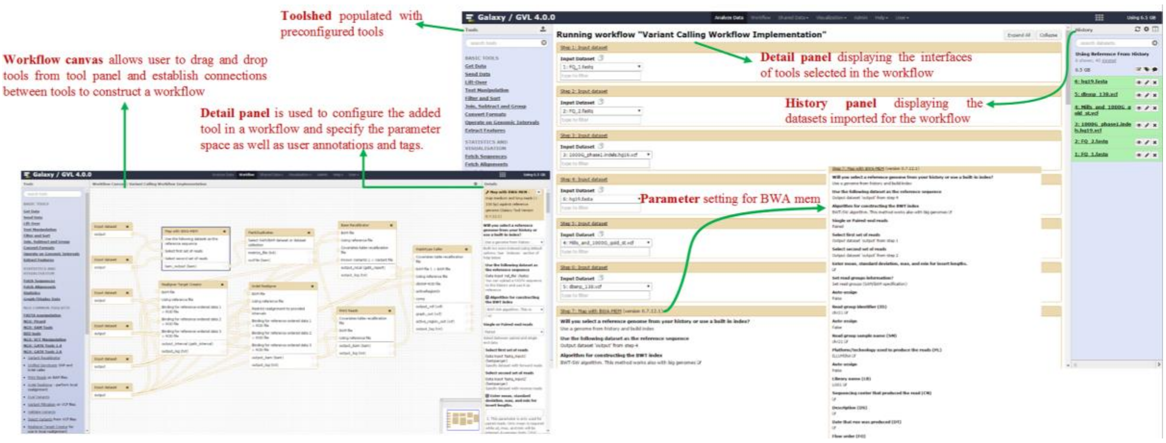

Figure 1 The representation of variant calling workflow on Galaxy interface. Workflow canvas was used to create the workflow from the tools populated in the tool shed. The parameter settings of the tools selected in the workflow were configured using the Detail panel. The compiled workflow is run using the data from the History panel. The interface of the tools was used to edit parameters (if required) and set input data for the tools at the runtime.

CWL:

A reference implementation of CWL designed for Python 2.7 was cloned and installed following the directions from the GitHub repository[1] manual. Implementing the GATK workflow in CWL required the development of a number of CWL definition files including

[1] https://github.com/common-workflow-language/cwltool

Yet Another Markup Language (YAML[2]) tool wrappers, JSON job files containing the input parameters and YAML test files for conformance tests; therefore produced for Picard MergeSAM, Picard SortSAM, Picard MarkDuplicates, GATK RealignTargetCreator, GATK IndelRealigner, GATK BaseRecalibrator, GATK PrintReads and GATK HaplotypeCaller as part of this study. In addition, the Docker images for BWA version[3] 0.7.12 and SAMtools version[4] 0.1.19 were already available on Docker hub. However to work with specific GATK (2.8) and Picard toolkit (1.136) versions, a new Docker image[5] was created and pushed to the Docker hub registry for public use.

A workflow in CWL consists of three main components: tool wrappers; input object; and a workflow file as shown in Figure 2.

A tool wrapper, as shown in Figure 2a, includes five main elements:

- File type and metadata: File type and metadata identifies tools as a CommandLineTool object and includes comments about the tool wrapper.
- Runtime environment: Runtime environment defines the execution environment of the tool and lists the requirements that must be fulfilled to allow successful execution of the tool.
- Input parameters: The input parameters define name and type of parameters and an “inputBinding” describes the actual command line argument.
- Output parameters: Similarly, the output parameters define name and type of parameters and an “inputBinding” describes the actual command line argument.
- Executable command: The actual execution command of the underlying tool is defined in the “baseCommand”. The input object associates input values with input parameters in the tool wrapper (Figure 2b).

Similarly the “outputs” section contains the list of output parameters comprising of the “source” that connects output parameters of the step (output_HaplotypeCaller) to the workflow output parameter. The HaplotypeCaller step depends on the output generated from the PrintReads step and the input parameter (inputbam_HaplotypeCaller) of the HaplotypeCaller step is connected to the output parameter (ouput_PrintReads) of the PrintReads step.

---

[2] http://www.yaml.org/
[3] https://hub.docker.com/r/scidap/bwa/
[4] https://hub.docker.com/r/isaacliao/samtools-0.1.19/
[5] https://hub.docker.com/r/fzkhan/picard-1.136-gatk-2.8/

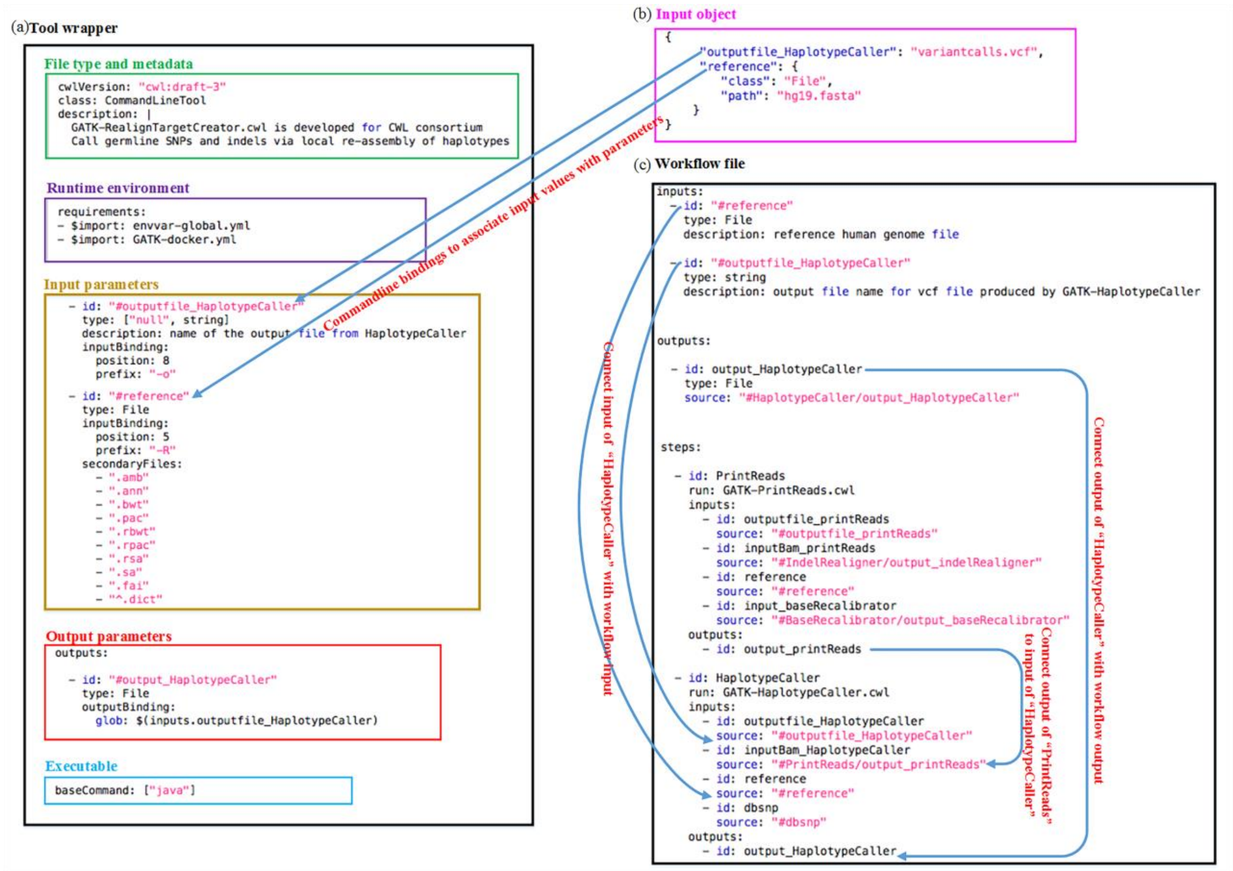

Figure 2 Snippets illustrating working and data flow in variant calling CWL workflow. (a) CWL tool wrapper describing tool specification for the HaplotypeCaller. (b) Input JSON object comprising of the parameter values. (c) CWL workflow file containing inputs, outputs and steps of the workflow

Cpipe:

Cpipe packages the older versions of the tools as compared to the Galaxy instance used in this analysis (Table 1). To ensure consistency, both pipelines were customized by ensuring similar versions for all the tools. In Galaxy, the latest update available for BWA was 0.7.12; hence BWA was updated to this version in both Galaxy and Cpipe (Figure 3).

Table 1 Default tool versions available in Galaxy in Cpipe

Toolkits	Galaxy	Cpipe
**BWA**	0.7.6	0.7.5a
**Picard tools**	1.136	1.65
**SAMtools**	0.1.19	0.1.19
**GATK**	2.8.1-g932cd3a	2.3.9

(a)

```
// Location of Picard tools here
PICARD_HOME="$BASE/tools/picard/picard-tools-1.65

// Due to GATK license restrictions, you must download
// GATK yourself and place it in this location
// (or point this to your installation)
// GATK="$TOOLS/gatk/2.3.9"
GATK="/mnt/galaxy/apps/cpipe_test/cpipe/tools/gatk/2.3.9"

// Location and version of BWA
BWA="$TOOLS/bwa/0.7.5a/bwa"
```

(b)

```
// Location of Picard tools here
PICARD_HOME="$BASE/tools/picard/picard-tools-1.136

// Due to GATK license restrictions, you must download
// GATK yourself and place it in this location
// (or point this to your installation)
// GATK="$TOOLS/gatk/2.3.9"
GATK="/mnt/galaxy/apps/cpipe_test/cpipe/tools/gatk/GenomeAnalysisTK-2.8-1-g932cd3a"

// Location and version of BWA
BWA="$TOOLS/bwa/bwa-0.7.12/bwa"
```

Figure 3 Screenshot of config file in Cpipe illustrating initialization of path variables to the toolkits. a) The default tool versions in the Cpipe config file. b) The default tool version updates, manual placement of toolkits in the respective directories and modification of config file to set the path variables to the location of new versions of toolkits in Cpipe.

In Cpipe, the Picard toolkit[6], BWA and Genome Analysis ToolKit (GATK) were updated to the latest versions available in Galaxy whereas the toolkit versions for SAMtools remained unchanged (Table 1). In addition, parameter settings (detailed in additional file 2) for each tool were also examined and made unified across both Cpipe and Galaxy instance.

Post-processing of result files:

The resultant vcf files from both the pipelines and truth set were filtered using the bed file [7] containing the chromosome 21 exome coordinates for comparison and evaluation over a consistent genomic coordinate set. Figure 4 shows the variants identified by Cpipe, Galaxy, CWL and their comparison with the known truth set.

---

[6] http://picard.sourceforge.net/

[7] https://swift.rc.nectar.org.au:8888/v1/AUTH_377/Paper-Data/InputData/broadinstitute-human-exomechr21.bed

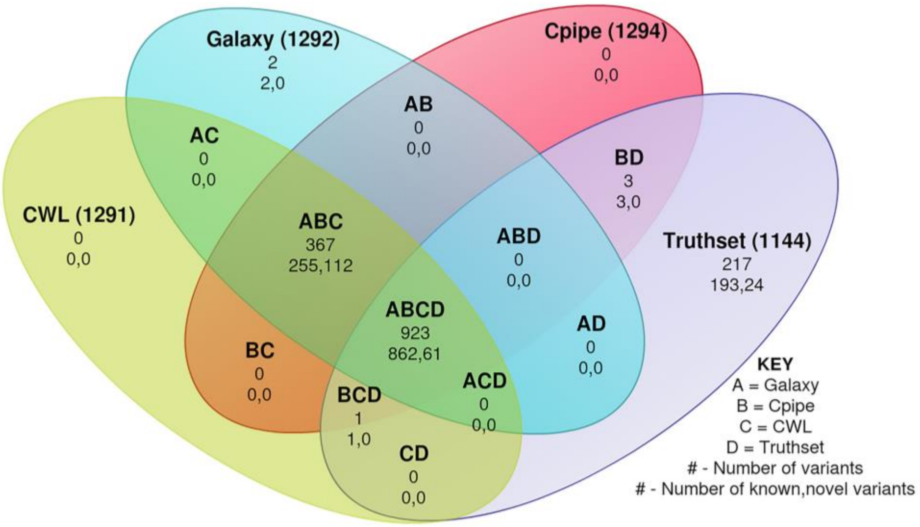

Figure 4 Each circle has total number of variants found only by that group and known versus novel variants. Venn diagram shows the comparison of results for Group A, B, C and D with similar pipeline components such as tool versions and parameter space. The Venn diagrams are generated using python script[8]

[8] https://github.com/Slugger70/vcf-venn
